# European Mitochondrial DNA Haplogroups are Associated with Cerebrospinal Fluid Biomarkers of Inflammation in HIV Infection

**DOI:** 10.20411/pai.v1i2.156

**Published:** 2016-11-01

**Authors:** David C. Samuels, Asha R. Kallianpur, Ronald J. Ellis, William S. Bush, Scott Letendre, Donald Franklin, Igor Grant, Todd Hulgan

**Affiliations:** 1 Vanderbilt Genetics Institute, Department of Molecular Physiology and Biophysics, Vanderbilt University, Nashville, Tennessee; 2 Genomic Medicine, Lerner Research Institute, Cleveland Clinic Foundation, Cleveland, Ohio; 3 University of California San Diego, San Diego, California; 4 Case Western Reserve University, Cleveland, Ohio; 5 Infectious Diseases, Vanderbilt University, Nashville, Tennessee

**Keywords:** HIV, Mitochondrial DNA, Tumor Necrosis Factor-alpha, Interleukin-6, Interleukin-8, Inflammation, Cerebral Spinal Fluid, Cytokines, Mitochondrial Haplogroups, Neuroinflammation

## Abstract

**Background:**

Mitochondrial DNA (mtDNA) haplogroups are ancestry-related patterns of single-nucleotide polymorphisms that are associated with differential mitochondrial function in model systems, neurodegenerative diseases in HIV-negative populations, and chronic complications of HIV infection, including neurocognitive impairment. We hypothesized that mtDNA haplogroups are associated with neuroinflammation in HIV-infected adults.

**Methods:**

CNS HIV Antiretroviral Therapy Effects Research (CHARTER) is a US-based observational study of HIV-infected adults who underwent standardized neurocognitive assessments. Participants who consented to DNA collection underwent whole blood mtDNA sequencing, and a subset also underwent lumbar puncture. IL-6, IL-8, TNF-α (high-sensitivity), and IP-10 were measured in cerebrospinal fluid (CSF) by immunoassay. Multivariable regression of mtDNA haplogroups and log-transformed CSF biomarkers were stratified by genetic ancestry using whole-genome nuclear DNA genotyping (European [EA], African [AA], or Hispanic ancestry [HA]), and adjusted for age, sex, antiretroviral therapy (ART), detectable CSF HIV RNA, and CD4 nadir. A total of 384 participants had both CSF cytokine measures and genetic data (45% EA, 44% AA, 11% HA, 22% female, median age 43 years, 74% on ART).

**Results:**

In analyses stratified by the 3 continental ancestry groups, no haplogroups were significantly associated with the 4 biomarkers. In the subgroup of participants with undetectable plasma HIV RNA on ART, European haplogroup H participants had significantly lower CSF TNF-α (*P* = 0.001).

**Conclusions:**

Lower CSF TNF-α may indicate lower neuroinflammation in the haplogroup H participants with well-controlled HIV on ART.

## INTRODUCTION

Central nervous system (CNS) immune surveillance is dysregulated in many neurodegenerative disorders, including HIV-associated neurocognitive disorder (HAND) [1–3]. Monocyte recruitment across the blood–brain barrier to the CNS is integral to this process. HIV infection of the CNS occurs soon after transmission, resulting in activation of monocytes and glial cells, and their ongoing recruitment to the CNS [4–6]. In addition to blood–brain barrier disruption, release of neurotoxic viral proteins (eg, HIV gp120, Tat, and Nef) from HIV-infected cells causes direct synaptodendritic damage, which is characteristic of HAND in the modern combination antiretroviral therapy (ART) era [7]. Ongoing neuronal injury due to persistent CNS inflammation and oxidative stress may promote HAND in the setting of HIV infection, despite effective viral suppression with ART [8, 9].

Cerebrospinal fluid (CSF) biomarkers are acceptable, albeit imperfect, surrogates for inflammation in brain tissue, and studies have reported pro-inflammatory cytokine and chemokine abnormalities in the CSF of HIV-infected adults even after ART initiation [10, 11]. Many cytokines are markers of monocyte and microglial activation [12–15]. Tumor necrosis factor alpha (TNF-α) and soluble TNF-α receptor levels are increased in persons with HIV-associated dementia (HAD) [10, 16–18]. Interferon *gamma*-induced protein 10 (IP-10, also referred to as CXCL10) is also elevated in the CSF of HIV-infected adults and correlates with cerebral metabolic patterns observed in HAND by magnetic resonance spectroscopy [19]. Significant variability in CSF inflammation has been suggested as a possible source of confounding in association studies of CSF cytokine levels with neurocognitive impairment [20].

Neurocognitive impairment and neuroinflammation are complex phenotypes and endopheno-types, respectively, in HIV-infected populations. Host genetic variants likely contribute to differences in phenotype expression and to relationships between endophenotypes and phenotypes. Monocytes/macrophages and lymphocytes that migrate into the CNS are major sources of inflammatory cytokines and chemokines during HIV infection, and these cells depend heavily on oxidative phosphorylation [21], a key mitochondrial function.

Patterns of variations in mitochondrial DNA (mtDNA) are used to define mitochondrial haplogroups, which have been shown to affect a range of HIV disease characteristics (for a review see [22]), including those potentially related to inflammation. *In vitro* studies have shown that European mitochondrial haplogroups differ in expression [23, 24] and methylation [25] of inflammation–pathway genes. A recent study from our group has reported significant associations between mitochondrial haplogroups and neurocognitive impairment in HIV-infected individual [26]. In this analysis, we explore the contribution of mtDNA haplogroups to inter-individual variability in CSF cytokine and chemokine levels as biomarkers of inflammation in the CNS. These CSF biomarkers have been previously linked to development of HAND [27, 28]. We therefore hypothesized that mtDNA haplogroups may be associated with differences in CSF inflammation, and performed planned subgroup analyses of haplogroups and CSF cytokine levels to explore these associations.

## METHODS

### Participants

CHARTER is a prospective, observational study of central and peripheral nervous system complications of HIV infection and treatment conducted at 6 US locations: Baltimore, Maryland; New York, New York; San Diego, California; Galveston, Texas; Seattle, Washington; and St. Louis, Missouri. Institutional review boards at each site approved the study, and each participant provided written informed consent. Data were collected between 2003 and 2007 according to a protocol of comprehensive neuromedical, neurobehavioral, and laboratory assessments that were standardized across sites [29]. The results reported herein are from a cross-sectional genetic association analysis of a subgroup of participants within CHARTER who underwent genetic studies and lumbar puncture for CSF sampling. All data utilized for these analyses were anonymized and de-identified.

As described previously [29], HAND categorization in CHARTER required a determination that neurocognitive and functional impairment were likely due to HIV-related effects on the brain rather than comorbid conditions. Detailed reviews by 2 senior CHARTER investigators, using published guidelines [30], provided categorization of comorbid conditions for all CHARTER participants as minimal, contributing, or confounding. Several conditions (eg, brain trauma, epilepsy, or other seizure history, CNS opportunistic diseases) informed this categorization; detailed information on their frequencies are presented elsewhere [29]. Individuals with confounding neurocognitive comorbidities (15% of the total CHARTER cohort), which by definition precluded an assessment of the contribution of HIV infection to their neurocognitive performance, were not eligible for a diagnosis of HAND according to Frascati criteria [29, 30]. Participants with confounding comorbidities were thus excluded from genetic analyses and hence from the present study. Though these analyses focused on neuroinflammatory biomarker endophenotypes rather than neurocognitive phenotypes, we elected to stratify analyses by minimal and contributing comorbidity status based on the possible contribution of these comorbidities (eg, diabetes, vascular disease, hepatitis C virus infection) to CSF biomarkers [31].

### Cytokine and Chemokine Measurement in CSF

Samples of CSF collected at entry from CHARTER participants were assayed for 4 cytokines: interleukin 6 (IL-6), interleukin 8 (IL-8), IP-10, and TNF-α. Cytokines were measured using commercially available immunoassays according to the instructions of the manufacturer, using high-sensitivity multiplex (IL-6, IL-8, and TNF-α) or standard (IP-10) bead-based immunoassay arrays (Luminex FLEXMAP 3D platform, Millipore, Billerica, Massachusetts). Ten percent of all assays were repeated to assess operator and batch consistency.

### Genetics

Isolation of DNA from whole blood samples was performed using PUREGENE (Gentra Systems Inc., Minneapolis, Minnesota). Full mtDNA sequencing was performed using the GeneChip Human Mitochondrial Resequencing Array 2.0 (Affymetrix, Inc., Santa Clara, California). Array intensity data were processed using the MitoChip Filtering Protocol (MFP) [32], variants were called relative to the revised Cambridge Reference Sequence (rCRS) [33] and haplogroups were assigned using the HaploGrep program (http://haplogrep.uibk.ac.at/) [34]. All participants also had nuclear DNA genotyping available using the Affymetrix Genome-Wide Human SNP Array 6.0 (Affymetrix, Inc., Santa Clara, California). Ancestry-informative markers were extracted from the autosomal DNA genotypes and were analyzed using EIGENSTRAT software [35] to generate principal components (PC). Clustering of PCs was used to define 3 common ancestry groups classified as European ancestry (EA), African ancestry (AA), and Hispanic ancestry (HA). Details of the clustering process are reported elsewhere [26]. Since mitochondrial haplogroups are closely related to continental ancestry [36], the analysis was stratified by the ancestry groups EA, AA, and HA.

### Statistics

Statistical tests included linear regressions of log-normalized cytokine levels. Participants were stratified into three groups based on PC-defined ancestry, as described previously. Within each common ancestry group, participants were assigned to the major mtDNA haplogroups, and statistical tests were carried out by comparing members of one haplogroup to all other members of that ancestral group (for example, haplogroup H vs. all other European ancestry participants). Regressions included the following covariates: sex, age (in years), dichotomized CSF HIV RNA load (≤ 50 copies/mL vs. > 50 copies/mL), whether participants were on ART, nadir CD4+ T-cell count as a continuous variable, and comorbidity classified as minimal to neurocognitive impairment or likely to contribute to but not confound the diagnosis of HAND (minimal vs. contributing) [29, 30]. Coding of dichotomous covariates used in the regressions is given in Supplemental Table 1. Outlier values were retained in the analysis, except in a sensitivity analysis described in the Results, in which outliers for IL-8 and TNF-α were removed. Statistical analyses were conducted using R version 2.15.1.

## RESULTS

**Supplemental Figure 1.**
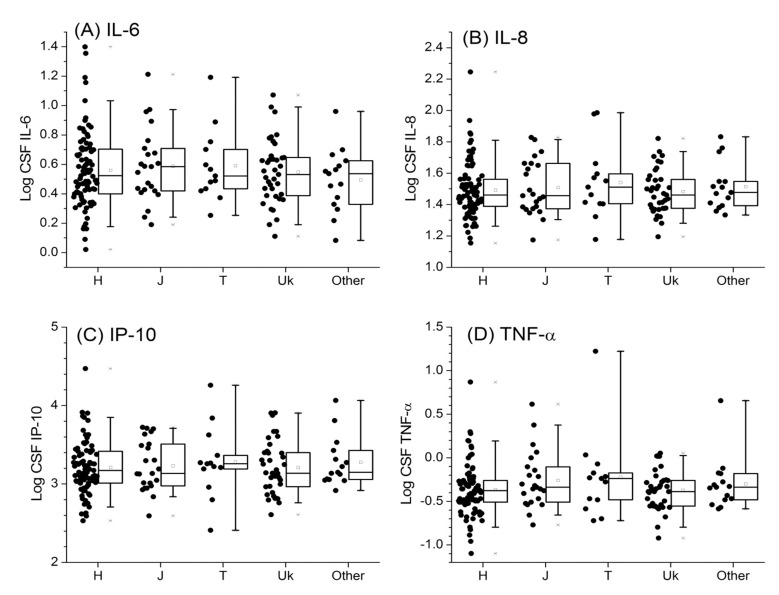
Measured cytokine levels in CSF for (A) IL-6, (B) IL-8, (C) IP-10, and (D) TNF-a in participants of European ancestry. Data are shown for each of the major European haplogroups: H, J, T, and Uk. All minor haplogroups are combined into the “other” category.

**Supplemental Figure 2.**
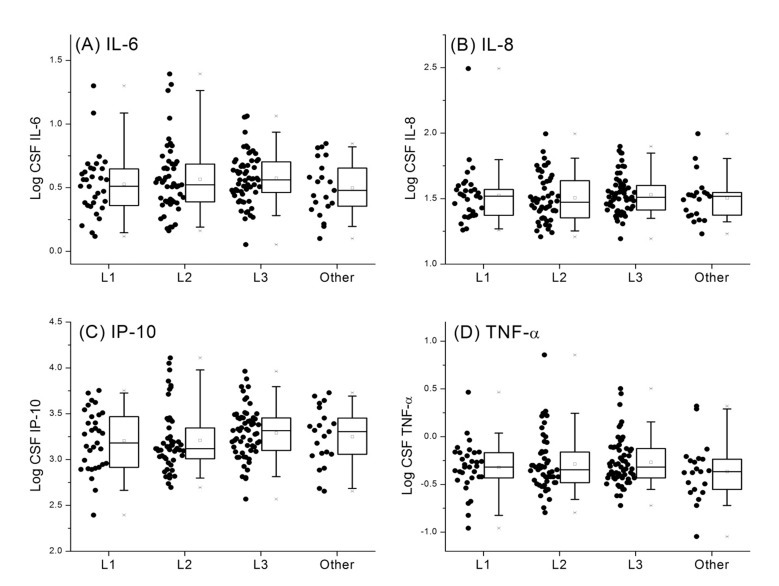
Measured cytokine levels in CSF for (A) IL-6, (B) IL-8, (C) IP-10, and (D) TNF-a in participants of African ancestry. Data are shown for the major African haplogroups L1, L2, and L3. All minor haplogroups are combined into the “other” category.

**Supplemental Figure 3.**
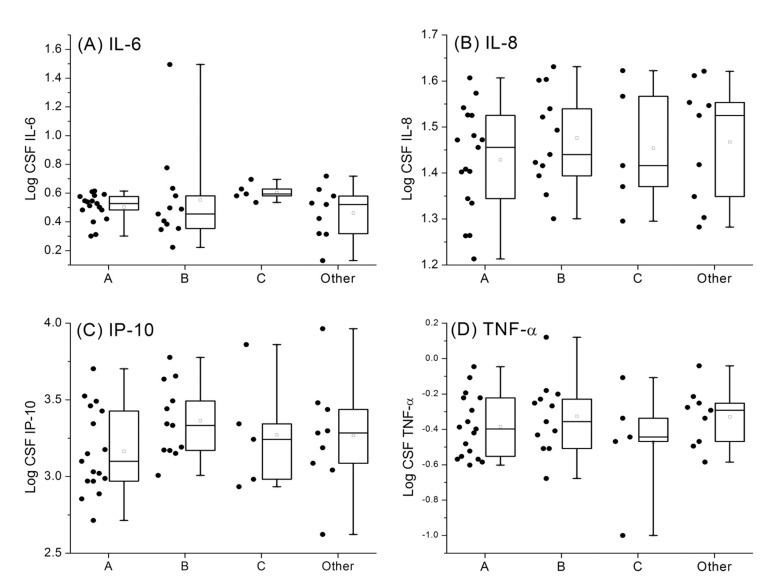
Measured cytokine levels in CSF for (A) IL-6, (B) IL-8, (C) IP-10, and (D) TNF-α in participants of Hispanic ancestry. Data are shown for the major Native American/Asian haplogroups A, B, and C found in persons of Hispanic ancestry. All minor haplogroups are combined into the “other” category.

A total of 384 participants had genetic data and CSF samples (Table 1); 45% were of European ancestry, 43% African ancestry, and 11% Hispanic ancestry. A majority (78%) were male, median age was 43 years, 74% were on ART at the time that CSF samples were collected, and 47% and 69% had plasma and CSF HIV RNA ≤ 50 copies/mL, respectively. Measured CSF cytokine levels are shown in Supplemental Figures 1–3 for the major mtDNA haplogroups within each of the 3 ancestral groups. Analyses included the major European haplogroups H, J, T, and Uk in participants of European ancestry (N = 174); African haplogroups L1, L2, and L3 in participants of African ancestry (N = 167); and for the Asian/Native American haplogroups, A and B in participants of Hispanic ancestry (N = 43). Linear regressions for the log-normalized CSF cytokine levels were calculated for each tested mtDNA haplogroup, adjusting for clinical and demographic covariates as described in the Methods section. None of the tested mtDNA haplogroups were significantly associated with the 4 measured CSF cytokine levels.

**Table 1.** Demographics of covariates for the 3 populations in the study.

	Total	European (EA)	African (AA)	Hispanic (HA)	P Value^a^
**Total N**	384	174	167	43	na
**Male**	300 (78%)	144 (83%)	120 (72%)	36 (84%)	0.31
					EA vs. AA 0.16
**Median Age [IQR], years**	43 [39–48]	44 [39–50]	43 [39–48]	40 [34–46]	EA vs. HA 0.0036
					AA vs. HA 0.033
**Contributing Comorbidity**	131	52 (30%)	61 (37%)	18 (42%)	0.23
					EA vs. AA 0.09
**Median Nadir CD4 [IQR]**	175 [52–308]	181 [74–330]	175 [35–283]	107 [52–240]	EA vs. HA 0.14
					AA vs. HA 0.83
**On ART**	283 (74%)	132 (76%)	118 (71%)	33 (76%)	0.49
**Plasma HIV RNA ≤50 copies/mL**	179 (47%)	90 (52%)	69 (41%)	20 (47%)	0.16
**CSF HIV RNA ≤50 copies/mL**	266 (69%)	131 (75%)	107 (64%)	28 (65%)	0.07

a Counts tested by 2 × 3 chi-square test; medians tested by Wilcoxon.

**Figure 1.**
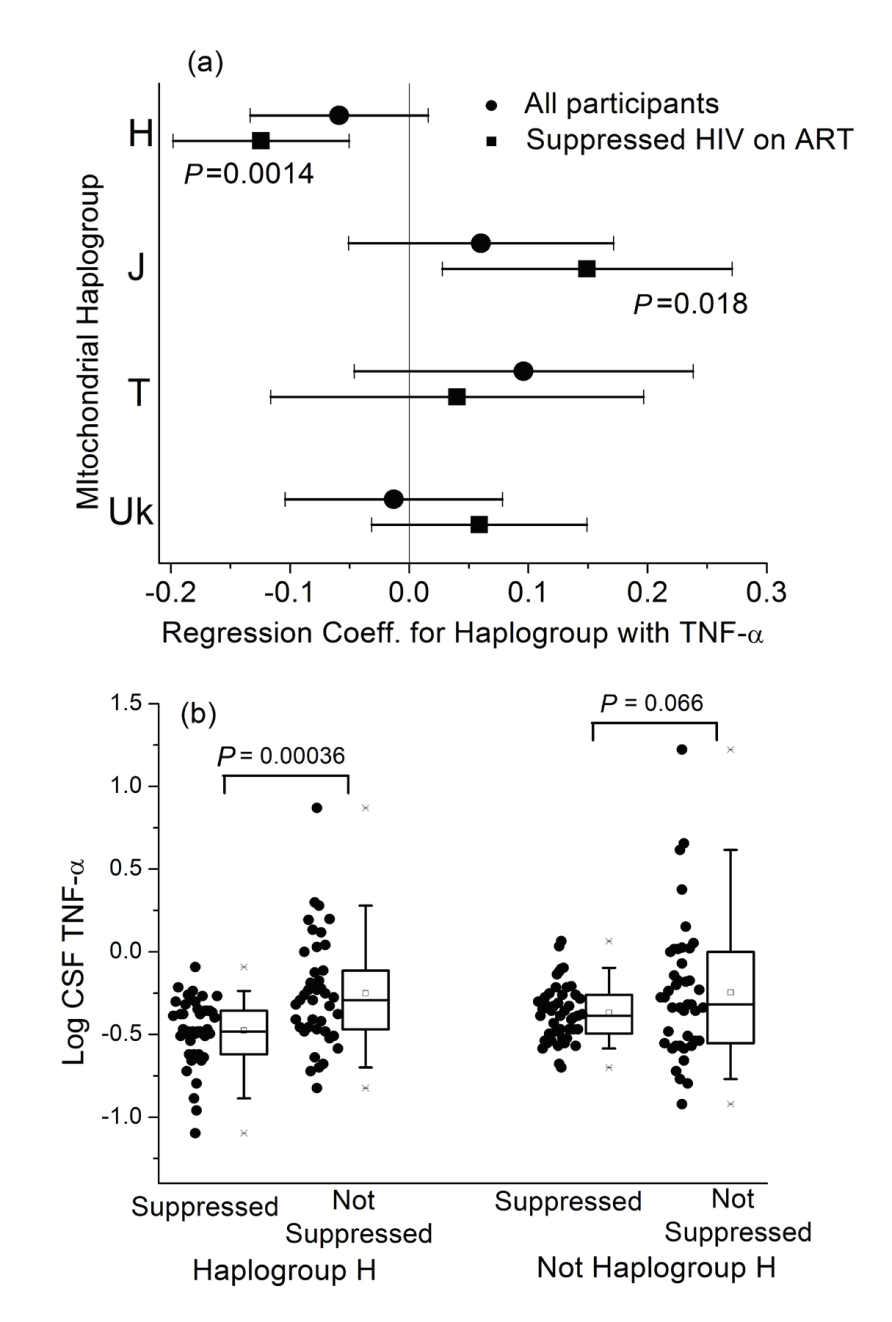
(A) Coefficients from the adjusted linear regression for log CSF TNF-α for the 4 European haplogroups tested. Results are shown for all participants (N = 174), and for the subset of patients on ART with HIV RNA levels ≤ 50 copies/mL (N = 90). *P* values are given for statistically significant regressions. (B) Univariate comparison of log CSF TNF-α values for Caucasian participants from Haplogroup H comparing participants with suppressed virus (plasma HIV RNA ≤ 50 copies/mL on ART) to nonsuppressed participants, and the same comparison in all other Caucasians.

Analyses were then restricted to participants on ART with plasma HIV RNA level ≤50 copies/mL (denoted as suppressed HIV on ART; Supplemental Tables 2–4). A small number of participants (3 of European ancestry and 2 of African ancestry) had detectable HIV RNA in their CSF though their HIV RNA in plasma was undetectable. These participants were retained in the analysis, and adjustment for detectable CSF HIV RNA was included in the analysis. Linear regression was carried out with the covariates described in Methods, with the exception of on/off ART, which was removed from the analysis. There were no significant associations of any of the 4 measured CSF cytokines with mitochondrial haplogroup in participants of African or Hispanic ancestry. In participants of European ancestry, the common haplogroup H had significantly lower CSF TNF-α levels (*P* = 0.001, Table 2, Figure 1A). For comparison, the results of this adjusted regression in the full Caucasian cohort are given in Supplemental Table 6. The lower CSF TNF-α level for haplogroup H participants with suppressed HIV also was significant in an unadjusted comparison (Figure 1B, *P* = 0.009 by *t* test). Among haplogroup H participants, individuals with suppressed HIV on ART showed significantly lower CSF TNF-α levels compared with those without suppression of HIV (*P* = 0.0004; Figure 1B). Among participants with other (non-H) European haplogroups, differences in log CSF TNF-α levels between individuals with and without suppressed HIV were smaller (but in the same direction as in Haplogroup H) and not statistically significant (*P* = 0.066).

**Table 2.** Multivariate linear regression results for log CSF TNF-α with mitochondrial haplogroup H for participants with undetectable HIV RNA on ART (N = 89).

	Regression beta	SE	*P* Value
**Haplogroup H vs All Others**	−0.12	0.04	0.0014
**Sex**	0.055	0.057	0.33
**Age (per year)**	0.0057	0.0024	0.019
**CSF HIV RNA Load ≤ 50 copies/mL**	−0.26	0.10	0.016
**Nadir CD4+ T Cell Count (per cell/mm^3^)**	−0.00015	0.00011	0.17
**Comorbidity Status (Minimal vs. Contributing)**	0.049	0.040	0.22

**Figure 2.**
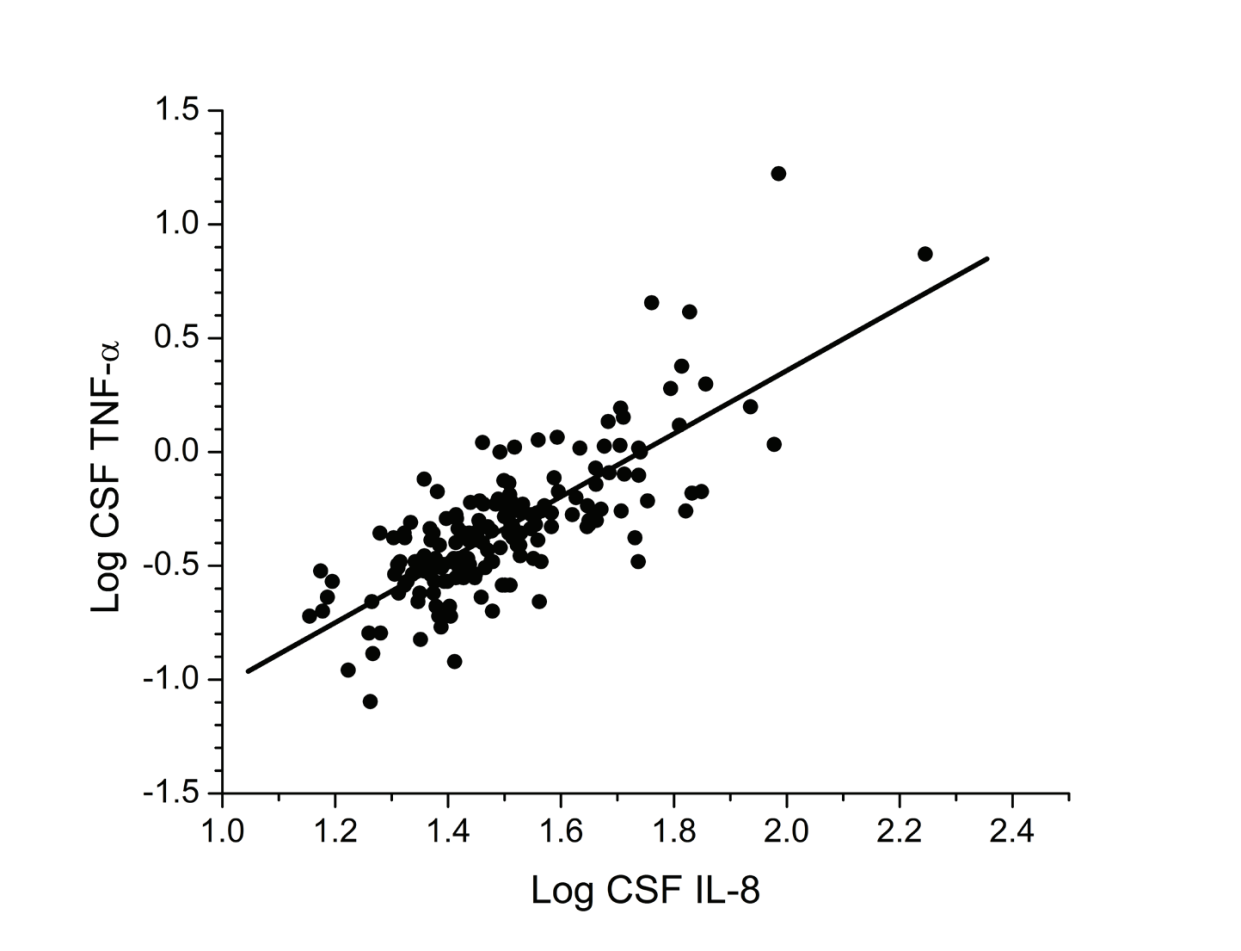
Comparison of log CSF IL-8 and log CSF TNF-α measurements in participants of European ancestry (R = 0.77; *P* < 0.0001).

The CSF TNF-α and CSF IL-8 values were highly correlated with each other (Figure 2). Therefore an association of haplogroup H with CSF TNF-α implies a similar association with CSF IL-8. Indeed, in the adjusted linear regression within participants with suppressed HIV on ART, haplogroup H did have a weak association with CSF IL-8 (regression beta = −0.05 ± 0.06, *P* = 0.085) in the same direction as that found for CSF TNF-α (Table 2).

Haplogroup J participants had higher TNF-α levels (*P* = 0.018, Table 3, Figure 1A), although this does not meet the multiple comparison-corrected significance threshold of *P* < 0.05 / 4 = 0.0125. Age and CSF HIV RNA detectability were significant covariates when modeling CSF TNF-α levels in both H and J haplogroup participants with suppressed HIV (Tables 2 and 3).

**Table 3.** Multivariate linear regression results for log CSF TNF-α with mitochondrial haplogroup J for participants with undetectable HIV on ART (N = 89).

	Regression beta	SE	*P* Value
Haplogroup J vs. All Others	0.15	0.06	0.018
Sex	0.058	0.059	0.32
Age (per year)	0.0068	0.0025	0.0082
CSF HIV RNA load ≤50 copies/mL	−0.26	0.11	0.019
Nadir CD4+ T Cell Count (per cell/mm^3^)	−0.00011	0.00011	0.30
Comorbidity Status (Minimal vs. Contributing)	0.032	0.041	0.43

We then stratified the analysis by the type of comorbidities present (ie, comorbid conditions deemed to be either minimal or contributing to neurocognitive deficits). The definitions of the minimal and contributing classification of comorbidities are given in the Participants section of the Methods previously mentioned, and references are cited there. Briefly, “minimal” and “contributing” comorbidities were those considered unlikely or possibly (respectively) to affect neurocognitive function. Linear regression was carried out using the covariates listed previously, with the exception of the comorbidity variable, which was removed. Again, no significant associations of mitochondrial haplogroup with the 4 cytokines emerged in African or Hispanic ancestry groups. In European ancestry participants with minimal comorbidities, haplogroup J participants had significantly higher TNF-α levels (*P* = 0.025, Supplementary Figure 3A) than those having other European haplogroups. In European ancestry participants with contributing comorbidities, both IL-8 and TNF-α levels were significantly higher in participants with haplogroup T (*P* = 0.005 and *P* = 0.0007, respectively, Supplemental Table 5, Supplementary Figure 3A, B). Although this effect is highly significant, this result must be interpreted with caution due to the small number of participants available for analysis after stratification. Of the 52 participants of European ancestry with contributing comorbidities, only 3 were classified as haplogroup T. It should also be noted that the IL-8 and TNF-α levels in CSF were highly correlated, so these are not independent associations (Figure 2). The participants with contributing comorbidities included 2 individuals with high IL-8 and TNF-α levels; one of these was from haplogroup T. When these 2 outliers were removed from the linear regression of the contributing comorbidity subset, the regression coefficient for haplogroup T for IL-8 dropped slightly to 0.23 [95% CI: 0.03–0.43] but remained significant (*P* = 0.023; compare with Supplemental Table 5). The regression coefficient for haplogroup T for TNF-α decreased to 0.24 [−0.03–0.52] and was no longer statistically significant (*P* = 0.092). It is also notable that these associations appear only in the subgroup of participants with contributing comorbidities. Although expert neurological assessments determined that these comorbidities would not confound neurocognitive function, it is possible that these same comorbidities could confound relationships between host factors and neuroinflammation biomarkers.

## DISCUSSION

In this analysis of HIV-infected adults in CHARTER, we observed ancestry-specific associations between mtDNA haplogroups and biomarkers of inflammation in CSF. These associations were independent of sex and age but were influenced by ART and HIV suppression, as well as comorbidities that may influence neurocognitive performance. To our knowledge, these are the first data to address this question.

Mitochondrial haplogroups are defined by patterns of variations in mtDNA that have accumulated over the past ~200,000 years [37]. Differences in fundamental functions of mitochondria across haplogroups have been reported [24, 38]. Mitochondrial haplogroups have been associated with many disease phenotypes [39, 40], including many phenotypes in HIV/AIDS [22]. One study [41] has shown that mitochondrial haplogroups can affect progression to AIDS. That study reported that the subgroup H3 (along with Uk and IWX) was significantly protective against progression to AIDS among HIV+ individuals. Although our study did not carry out any analysis at the sub-haplogroup level, H3 is typically a major component of the H haplogroup population, and we found that haplogroup to have lower CNS TNF-α levels, with the strongest effect in HIV+ participants with suppressed HIV on ART (Figure 1). TNF-α is a pro-inflammatory biomarker associated with increased viral replication [42–44]. Thus the influence of H3 on disease progression might be mediated by TNF-α expression levels. Most relevant for our study, a considerable body of recent work has shown that neuroinflammation is closely related to mitochondrial dys-function [45–48]. The general conclusion from these studies has been that mitochondrial function and neuroinflammation interact through nitric oxide and reactive oxygen species production by mitochondria, which contribute to further neurodegeneration and enhanced neuroinflammation in a positive feedback loop.

Although there are no well-established clinical biomarkers of neuroinflammation or neurocognitive performance in the setting of HIV disease and ART, observed associations with IL-8 and TNF-α may be clinically relevant. Prior studies have reported elevated IL-8 in CSF of HIV-infected adults with undetectable plasma HIV RNA on ART regardless of cognitive status [49]. A combination of MCP-1 and TNF-α correctly classified CHARTER participants with stable neuro-cognitive impairment (NCI) [50]. In our analysis, the group of participants with suppressed HIV on ART, the median CSF TNF-α level in haplogroup H participants was 19% lower than in all other European ancestry participants (Figure 1B).

Mitochondrial DNA variants are increasingly recognized as influencing mitochondrial function [51, 52], particularly in energetically vulnerable tissues like the CNS [53, 54]. Given the prevalence of neurodegenerative phenotypes in inherited mitochondrial diseases, the centrality of mitochondrial function in cellular energy production, oxidative stress, and apoptotic regulation, a connection between neuroinflammation and mtDNA variants is biologically plausible. In recent CHARTER analyses, we identified an association between the mtDNA haplogroup B and better neurocognitive performance among Hispanic ancestry participants [34]. We did not see differences in the measured cytokines based on mtDNA haplogroups in the Hispanic population, perhaps due to the smaller sample size in the cytokine dataset compared with the NCI analysis, or perhaps because other pathways or biomarkers are involved in these different phenotypes. The small sample size of the Hispanic population in this study (Table 1) means that no definitive conclusion can be reached in this study about the lack of association of mitochondrial haplogroups with CSF biomarker levels. Future analyses should further investigate the Hispanic population with a larger population and broader range of CSF biomarkers. Conversely, although our previous analysis did not find associations between mtDNA haplogroups and NCI or HAND in participants of European or African ancestry, we do find significant differences in the cytokine measures in the European ancestry participants.

Strengths of these analyses include batched assays on carefully collected and cryopreserved CSF, and the use of high-sensitivity assays. In addition, full mtDNA sequence data allowed for optimal haplogroup determination, and available genome-wide data provided ancestry-informative markers for genetic ancestry determination. The CHARTER cohort includes very well-characterized phenotyping using standardized neuropsychometric methods, and persons with neurologic comorbidities that could confound the interpretation of neurocognitive information were excluded from our analyses.

Limitations of this study include the relatively small sample sizes of individual mtDNA haplogroups after stratification for ancestry, comorbidity, and or ART status. Nonetheless, to our knowledge, this is the largest sample with combined CSF biomarker and mtDNA genetic data, and several potential associations remained statistically significant after applying conservative corrections for multiple statistical comparisons. Although analyses were either adjusted or stratified for potential confounding factors, the CHARTER population is heterogeneous, and there may be confounders that remain unmeasured or unaccounted for, which could have influenced the observed associations. We chose to adjust for plasma viral loads as a dichotomous variable (with a threshold at 50 copies/mL). We cannot exclude the possibility that variations in the plasma viral load above 50 copies/mL might affect our observations. With the available data from CHARTER participants and this cross-sectional analysis, we are unable to draw conclusions about causality or mechanisms.

Future studies should include prospective, targeted assessments of neuroinflammation before and after ART in persons of particular ancestry and haplogroups, and *in vitro* experiments in cell and or animal model systems to characterize genetic variation in neuropathogenesis. More extensive analyses of mtDNA sequence can be undertaken to determine if additional associations are seen with non-haplogroup-associated variants, or in particular mitochondrial gene regions.

## Figures and Tables

**Supplementary Figure 4.**
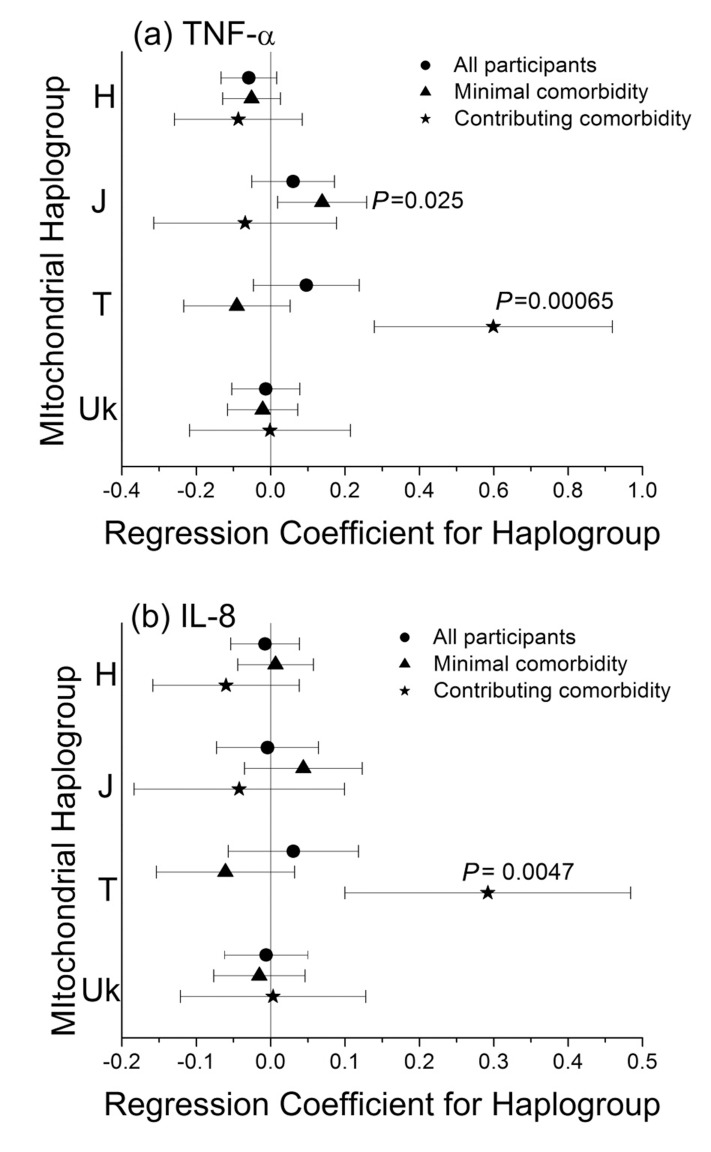
Regression coefficients for Log CNS TNF-α (A) and log CNS IL-8 (B). Results are shown for all European ancestry participants (N = 174), and then stratified by comorbidity type (minimal [N = 122] or contributing [N = 52]) for neurocognitive impairment.

## Appendix

**Supplementary Table 1.** Coding of covariates for regressions

	Value = 0	Value = 1
**Sex**	Female	Male
**CSF Viral RNA Load**	> 50 copies/mL	≤ 50 copies/mL
**Comorbidity**	Minimal	Contributing
**On ART**	No	Yes

**Supplementary Table 2.** Distributions of the populations for the secondary analyses in the participants of European ancestry, broken out by major haplogroups H, J, T, and Uk.

	H	J	T	Uk	Other
**Total**	85 (49%)	23 (13%)	13 (7%)	38 (22%)	15 (9%)
**Contributing Comorbidity**	28 (33%)	8 (35%)	3 (23%)	10 (26%)	3 (20%)
**Plasma HIV RNA ≤50 copies/mL on ART**	43 (51%)	10 (43%)	6 (46%)	22 (58%)	8 (53%)

**Supplementary Table 3.** Distributions of the populations for the secondary analyses in the participants of African ancestry, broken out by major haplogroups L1, L2, and L3.

	L1	L2	L3	Other
**Total**	32 (19%)	52 (31%)	62 (37%)	21 (13%)
**Contributing Comorbidity**	7 (22%)	21 (40%)	27 (44%)	6 (29%)
**Plasma HIV RNA ≤50 copies/mL on ART**	11 (34%)	22 (42%)	28 (45%)	6 (29%)

**Supplementary Table 4.** Distributions of the populations for the secondary analyses in the participants of Hispanic ancestry, broken out by major haplogroups A, B, and C.

	A	B	C	Other
**Total**	17 (40%)	12 (28%)	5 (12%)	9 (21%)
**Contributing Comorbidity**	10 (59%)	2 (17%)	4 (80%)	2 (22%)
**Plasma HIV RNA ≤50 copies/mL on ART**	7 (41%)	7 (58%)	1 (20%)	4 (44%)

**Supplemental Table 5.** Multivariate linear regression results for log CSF IL8 and TNF-α with mitochondrial haplogroup T for participants with contributing morbidity for neurocognitive impairment.

	Log CSF IL-8			Log CSF TNF-a		
	beta	SE	*P* Value	beta	SE	*P* Value
**Haplogroup T vs. All Others**	0.29	0.10	0.0047	0.60	0.16	0.00065
**Sex**	0.086	0.067	0.20	−0.002	0.112	0.99
**Age (per year)**	0.0014	0.0031	0.67	−0.6e–3	5.2e–3	0.91
**CSF HIV RNA Load ≤50 copies/mL**	−0.22	0.11	0.049	−0.32	0.18	0.082
**On ART**	−0.04	0.11	0.69	−0.11	0.18	0.53
**Nadir CD4+ T Cell Count (per cell/mm^3^)**	−1.4e–4	1.8e–4	0.41	−0.6e–4	3.0e–4	0.85

**Supplemental Table 6.** Multivariate linear regression results for log CSF TNF-α with mitochondrial haplogroup H for all Caucasian participants (N = 174).

	Regression beta	SE	*P* Value
**Haplogroup H vs. All Others**	−0.059	0.038	0.12
**Sex**	0.037	0.059	0.53
**Age (per year)**	0.0081	0.0023	0.00056
**CSF HIV RNA Load ≤50 copies/mL**	−0.26	0.06	8.9e–5
**Nadir CD4+ T Cell Count (per cell/mm^3^)**	−0.00026	0.00010	0.012
**Comorbidity Status (Minimal vs. Contributing)**	0.074	0.042	0.079
**On ART**	−0.196	0.070	0.006
